# Absorbable Phenylpropenoyl Sucroses from *Polygala tenuifolia*

**DOI:** 10.3390/molecules16075507

**Published:** 2011-06-29

**Authors:** Gaimei She, Yinying Ba, Yang Liu, Hang Lv, Wei Wang, Renbing Shi

**Affiliations:** School of Chinese Pharmacy, Beijing University of Chinese Medicine, Beijing 100102, China; Email: shegaimei@126.com (G.S.); byy3333@sina.com (Y.B.); netug@126.com (Y.L.); lvhang87@hotmail.com (H.L.); hplc_ms@126.com (W.W.)

**Keywords:** absorbable phenylpropenoyl sucrose, sibiricose A5, sibiricose A6, 3′,6-disinapoyl sucrose, *Polygala tenuifolia*

## Abstract

Three phenylpropenoyl sucroses – sibiricose A5 (**1**), A6 (**2**) and 3′,6-disinapoyl sucrose (**3**) – were isolated from the 30% EtOH extract of *Polygala tenuifolia*, which displayed antidepressant-like action. HPLC analysis indicated that the three phenylpropenoyl sucroses could be absorbed into serum. From the serum pharmacochemistry point of view, these three phenylpropenoyl sucroses might prevent or relieve depression.

## 1. Introduction

*Polygala tenuifolia* (local name: Yuan zhi) is widely distributed in China, and its root is an important herb used in Traditional Chinese Medicine to treat depression. A number of investigations had been carried out to identify the compounds responsible of the biological activity of Yuan zhi [1,2,3,4]. Some phenylpropenoyl sucroses from Yuan zhi became hot research topics because of their bioactivity of preventing memory disorders [5,6,7,8]. The understanding and diagnosis of many diseases mainly relies on the metabolic profiling of biological fluids such as urine and plasma, as their compositions reflect the biochemical status of a living organism and imply information about the biological processes associated with pathological conditions. The application of high-performance liquid chromatography (HPLC) for untargeted plasma metabolic profiling with direct comparison with authentic samples in metabonomic research has attracted much attention in recent years [9,10]. In order to research the untargeted plasma metabolite profiling of phenylpropenoyl sucroses, we examined the metabolites in rat serum using HPLC by direct comparison with authentic samples after the rats are administered orally with Yuan zhi aqueous extracts. As a result, sibiricose A_5_ (**1**), A_6_ (**2**) and 3′,6-disinapoyl sucrose (**3**) were absorbed into serum, as we report here for the first time. 

## 2. Results and Discussion

### 2.1. Identification of Compounds 1 to 3

The 30% EtOH extract of Yuan zhi exhibited more notable activity in the antidepressive assay than the 50%, 70% and 90% EtOH extracts [11]. The proportion of phenylpropenoyl sucroses in the 30% EtOH fraction of Yuan zhi is 44.88%, which is calculated by their peak area ratio in HPLC-DAD. Thus, we focus on the phenylpropenoyl sucroses-enriched fraction in this study. 

**Figure 1:**
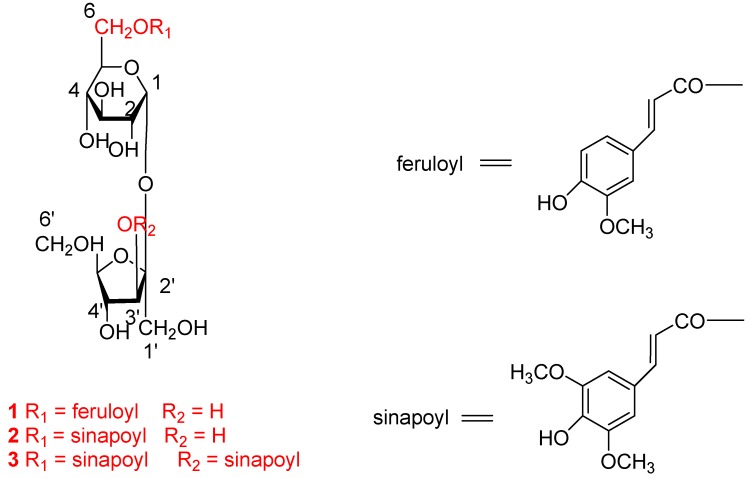
Structures of compounds **1**-**3**.

Repeated column chromatography (CC) over Sephadex LH-20 and silica gel column led to the isolation of three phenylpropenoyl sucroses **1**-**3** (Figure 1) from this fraction. These compounds were determined to be sibiricose A_5_ (**1**) [12], A_6_ (**2**) [12] and 3′,6-disinapoyl sucrose (**3**) [13], respectively, by comparison of their spectroscopic data with reported literature values and authentic samples. The full NMR assignments of **1**, **2** and **3** (Table 1) were achieved by the detailed 2D NMR analysis.

Reference samples of sibiricose A5 (**1**), A6 (**2**) and 3′,6-disinapoyl sucrose (**3**) were prepared by repeated CC on Sephadex LH-20, ODS-A, silica gel and RP-HPLC of the three crude samples.

**Table 1:** ^1^H-NMR (500 and 400 MHZ) and ^13^C-NMR (125 and 100 MHz) data of compounds **1**-**3** in CDCl_3 _(*δ* in ppm, *J* in Hz).

Position	1		2		3	
	δ_C_	δ_H_	δ_C_	δ_H_	δ_C_	δ_H_
Glycone moiety						
Glc-1	93.3	5.36 (d, 4)	93.3	5.37 (d, 4)	92.9	5.50 (d, 3.7)
2	73.1	3.53 (d, 10.0, 4.0)	73.1	3.51 (d, 10.0, 4.0)	73.1	3.50 (m)
3	74.9	3.69 (dd, 10.0, 9.0)	75.0	3.75 (dd, 10.0, 9.5)	75.0	3.69 (t, 9.3)
4	71.2	3.38 (dd, 9.0, 9.0)	71.2	3.34 (dd, 9.5, 9.0)	71.9	3.38 (m)
5	73.8	3.88 (m)	74.5	3.89 (m)	72.8	4.29 (m)
6	62.3	3.77 (dd, 12.0, 4.5)3.81 (m)	62.4	4.31 (dd, 12.0, 4.5)4.52 (m)	65.6	4.48 (br d, 10.0)4.06 (m)
Fru-1	65.4	3.60 (d, 12.0)	65.4	3.62 (d, 12.0)	65.4	3.60 (d, 12.0)
		3.69 (d, 12.0)		3.65 (d, 12.0)		3.63 (d, 12.0)
2	104.4		104.8		104.8	
3	79.7	5.40 (d, 8.0)	79.6	5.46 (d, 8.0)	79.6	5.58 (d, 7.5)
4	74.8	4.33 (dd, 8.0, 7.5)	74.5	4.07 (dd, 8.0, 7.5)	74.4	4.51 (t, 8.0)
5	84.1	3.88 (m)	84.2	3.88 (m)	84.2	4.10 (m)
6	62.9	3.84 (m)	62.9	3.89 (m)	63.7	3.99 (m)
		3.84 (m)		3.80 (m)		4.20 (m)
Aglycone moiety						
R_1_-1	127.5		129.5		125.1	
2	112.1	6.74 (d, 2.0)	107.1	6.90 (s)	106.8	7.12 (s)
3	149.6		149.5		149.0	
4	149.0		130.7		140.5	
5	116.5	6.68 (d, 8.0)	149.5		149.0	
6	124.3	6.59 (dd, 8.0, 2.0)	107.1	6.90 (s)	106.8	7.12 (s)
7	147.7	7.71 (d, 15.6)	147.2	7.64 (d, 15.6)	146.0	8.09 (d, 16.0)
8	115.1	6.36 (d, 15.6)	115.8	6.38 (d, 15.6)	115.2	6.90 (d, 16.0)
9	168.3		169.1		166.8	
-OCH_3_	56.6	3.86 (s)	56.8	3.87 (s)	56.4	3.80 (s)
R_2_-1					125.2	
2					106.9	7.12 (s)
3					149.2	
4					140.5	
5					149.2	
6					107.9	7.12 (s)
7					146.7	7.98 (d, 15.6)
8					115.6	6.67 (d, 15.6)
9					167.7	
-OCH_3_					56.9	3.80 (s)

### 2.2. Untargeted Plasma Metabolite Profiling of Yuan zhi

**Figure 2:**
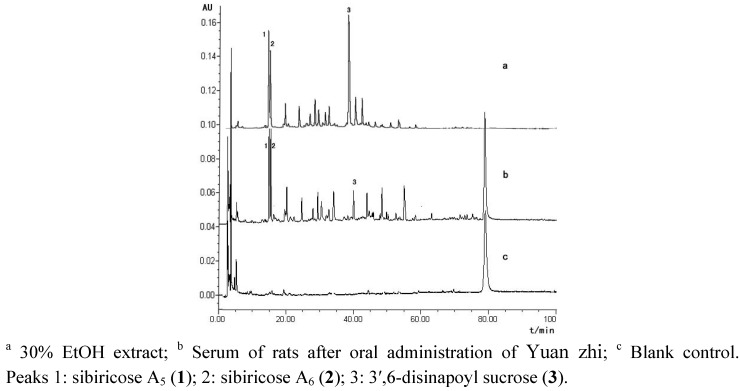
Plasma metabolite HPLC profiles of *Yuan zhi *extract.

The aqueous extract of *P*. *tenuifolia* (2.5 g crude drug/mL) was administered orally to male Sprague-Dawley (SD) rats at a dose of 1.56 g/kg. The orbital blood samples (3 mL) of the rats were collected at 60 min post-dose. The serum containing the extracts of *P*. *tenuifolia *and blank control serum were analyzed by HPLC at 330 nm after a series of preconditioning (Figure 2). As shown in the Figure, HPLC analysis indicated that three phenylpropenoyl sucroses sibiricose A5 (**1**), A6 (**2**) and 3′,6-disinapoyl sucrose (**3**) of Yuanzhi, can be absorbed into serum. Our feasibility study showed the proportions of sibiricose A5 (**1**), A6 (**2**) and 3′,6-disinapoyl sucrose (**3**) in the fraction were 11.06%, 8.89%, 24.29%, respectively, by calculating their peak area ratio in HPLC-DAD. It was also reported that the fraction containing these three oligosaccharide polyesters as main chemical constituents had obvious antidepressant activity [11]. 

In addition, it is reported that 3′,6-disinapoyl sucrose (**3**) can protect the SH-SY5Y human neuroblastoma cells from lesion, and sibiricose A5 (**1**) can protect the PC12 cells from the damage induced by glutamic acid [11,14]. Therefore, from the point of view of serum pharmacochemistry, our results indicate that the three phenylpropenoyl sucroses might prevent or relieve depression.

## 3. Experimental

### 3.1. General

Column chromatography (CC) was performed on Sephadex LH-20 (Pharmacia Fine Chemical Co., Ltd.), SP825 (Mitsuishi Chemical Co.), Chromatorex ODS (Fuji Silysia Chemical Co., Ltd.) and silica gel (Qingdao Haiyang Chemical Co.). TLC was carried on silica gel G precoated plates (Qingdao Haiyang Chemical Co.) with CHCl_3_-MeOH-H_2_O (9:1:0.1 or 7:3:0.5). The spots were detected by spraying with 10% H_2_SO_4_ ethanol solution followed by heating. ^1^H- and ^13^C-NMR, HSQC and HMBC spectra were recorded in DMSO-d_6_ with Bruker AM-400 and DRX-500 spectrometers. The HPLC was performed with a Waters 2695 HPLC system on a Agela Venusil MP-C18 column (5 μm, 250 × 4.6 mm, Agela, China) and with column temperature set at 30 °C, using a diode-array detection (Shimadzu SPD-M10A). 

### 3.2. Plant Material

The roots of *P*. *tenuifolia* were collected from Shanxi province, China, and identified by Yuting Chen (Beijing University of Chinese Medicine).

### 3.3. Extraction and Isolation

Dried plant material (300 g) of *P*. *tenuifolia* was refluxed with ethanol for 3 h (4 times). After removal of the organic solvent under reduced pressure, the aqueous fraction was concentrated to a small volume (100 mL) and subjected to a SP825 macroporous adsorptive resin column eluting with H_2_O and 30% EtOH to afford two fractions. The concentrated 30% EtOH was applied to CC on silica gel (CHCl_3_-MeOH-H_2_O, 9:1:0.1-7:3:0.5), Sephadex LH-20 and ODS-A, eluting with H_2_O-MeOH (1:0-0:1) to afford compounds **1** (14 mg), **2** (6 mg) and **3** (15 mg), respectively.

### 3.4. HPLC Analysis

The plasma sample (0.5 mL) was placed in a 5 mL polypropylene tube, and monopotassium phosphate (0.2 mL) and acetonitrile (1.5 mL) were added to the tube. The tube was vortexed-mixed for 90 s. The precipitated protein was removed by centrifugation at 4,000 rpm for 30 min. The organic layer was transferred to another tube, and evaporated to dryness by N_2_ at 50 °C. The residue was added 0.2 mL distilled water to dissolve, and then filtered through a 0.45 μm Millipore filter film. Aliquots of 20 μL were injected into the HPLC system. The mobile phase consisted of A, CH_3_CN; B, H_2_O (0.1% phosphoric acid); gradient: 11-16% A linear in 10 min; 16-22% A linear in 10 min; 22-23% A linear in 5 min; 23-28% A linear in 9 min; 28% A linear in 11 min; 28-33% A linear in 10 min; 33-39% A linear in 15 min; 39-42% A linear in 15 min; 42-55% A linear in 10 min; 55-70% A linear in 5 min; isocratic on 70-11% A for 10 min. The mobile phase was degassed automatically using the electronic degasser system. The flow rate was 1.0 mL/min. The optimum wavelength was set at 330 nm [15].

### 3.5. Animal Experiments

Male SD rats (250 ± 20 g body weight) were provided by Vital River Experimental Animal Co., Ltd. (license number: SCXK2006-0009). Rats were kept in an environmentally controlled breeding room for three days before starting the experiment and were fed with standard laboratory food and water. SD rats had free access to water but no food for 12 h before the experiments. The extract of Yuan zhi was administered orally to rats at a dose of 1.56 g/kg, and the blood samples (3 mL) were collected at 60 min post-dose. The plasma samples were immediately separated by centrifugation at 4,000 rpm for 10 min.
